# Comprehensive Characterization of the Multiple Myeloma Immune Microenvironment Using Integrated scRNA-seq, CyTOF, and CITE-seq Analysis

**DOI:** 10.1158/2767-9764.CRC-22-0022

**Published:** 2022-10-25

**Authors:** Lijun Yao, Reyka G. Jayasinghe, Brian H. Lee, Swati S. Bhasin, William Pilcher, Deon Bryant Doxie, Edgar Gonzalez-Kozlova, Surendra Dasari, Mark A. Fiala, Yered Pita-Juarez, Michael Strausbauch, Geoffrey Kelly, Beena E. Thomas, Shaji K. Kumar, Hearn Jay Cho, Emilie Anderson, Michael C. Wendl, Travis Dawson, Darwin D'souza, Stephen T. Oh, Giulia Cheloni, Ying Li, John F. DiPersio, Adeeb H. Rahman, Kavita M. Dhodapkar, Seunghee Kim-Schulze, Ravi Vij, Ioannis S. Vlachos, Shaadi Mehr, Mark Hamilton, Daniel Auclair, Taxiarchis Kourelis, David Avigan, Madhav V. Dhodapkar, Sacha Gnjatic, Manoj K. Bhasin, Li Ding

**Affiliations:** 1 Washington University School of Medicine, Saint Louis, Missouri.; 2 Icahn School of Medicine at Mt. Sinai, New York, New York.; 3 Emory University, Atlanta, Georgia.; 4 Mayo Clinic, Rochester, Minnesota.; 5 Beth Israel Deaconess Medical Center, Harvard Medical School, Boston, Massachusetts.; 6 Multiple Myeloma Research Foundation, Norwalk, Connecticut.

## Abstract

As part of the Multiple Myeloma Research Foundation (MMRF) immune atlas pilot project, we compared immune cells of multiple myeloma bone marrow samples from 18 patients assessed by single-cell RNA sequencing (scRNA-seq), mass cytometry (CyTOF), and cellular indexing of transcriptomes and epitopes by sequencing (CITE-seq) to understand the concordance of measurements among single-cell techniques. Cell type abundances are relatively consistent across the three approaches, while variations are observed in T cells, macrophages, and monocytes. Concordance and correlation analysis of cell type marker gene expression across different modalities highlighted the importance of choosing cell type marker genes best suited to particular modalities. By integrating data from these three assays, we found International Staging System stage 3 patients exhibited decreased CD4^+^ T/CD8^+^ T cells ratio. Moreover, we observed upregulation of *RAC2* and *PSMB9*, in natural killer cells of fast progressors compared with those of nonprogressors, as revealed by both scRNA-seq and CITE-seq RNA measurement. This detailed examination of the immune microenvironment in multiple myeloma using multiple single-cell technologies revealed markers associated with multiple myeloma rapid progression which will be further characterized by the full-scale immune atlas project.

**Significance:**

scRNA-seq, CyTOF, and CITE-seq are increasingly used for evaluating cellular heterogeneity. Understanding their concordances is of great interest. To date, this study is the most comprehensive examination of the measurement of the immune microenvironment in multiple myeloma using the three techniques. Moreover, we identified markers predicted to be significantly associated with multiple myeloma rapid progression.

## Introduction

Single-cell sequencing technologies offer advantages over traditional bulk methods in cancer genomics research for evaluating cellular heterogeneity and investigating evolution of cellular subpopulations between the tumor and its microenvironment. For example, single-cell methods have been extensively applied to multiple myeloma, a highly heterogeneous disease marked by uncontrolled clonal expansion of plasma cells. Single-cell RNA sequencing (scRNA-seq) has been used to examine tumor and immune cell populations (1, 2) and mass cytometry (CyTOF) to evaluate the impact of drugs on immune populations in multiple myeloma (3). The third technology, cellular indexing of transcriptomes and epitopes by sequencing (CITE-seq), is a more recent, multimodal approach with simultaneous quantification of single-cell transcriptomes and surface proteins. All three approaches enable identification of cell types, cell states, and characterization of cellular heterogeneity at transcriptomic and/or protein levels. Consequently, understanding their concordances across technologies is of great practical interest.

In addition, the bone marrow microenvironment plays an important role in the evolution of premalignant multiple myeloma, multiple myeloma progression, and treatment response. Single-cell transcriptomics analysis of the tumor microenvironment (TME) revealed compositional alterations begin at the monoclonal gammopathy of undetermined significance (MGUS) stage, including enrichment of T cells, natural killer (NK) cells, and CD16^+^ monocytes (2). Specifically, the percentage of CD4^+^ T cells was significantly reduced in bone marrow of patients with multiple myeloma, leading to altered CD4^+^ T/CD8^+^ T ratio (4). When comparing the clinical status, the ratio decreased in International Staging System (ISS) stage 3 patients compared with stage 1 patients (5). With respect to treatment, the proportion of CD3^+^ T cells was lower in treated patients compared with patients with chemo-naïve multiple myeloma (6). Further work is needed to expand initial findings using various assays and reveal candidate markers for characterizing clinical features of patients with multiple myeloma and optimizing treatment.

Combining the timeliness of the technology concordance question with furtherance of multiple myeloma research, we subjected bone marrow samples from 18 patients with multiple myeloma to scRNA-seq, CyTOF, and CITE-seq, examining the similarities across the aforementioned single-cell techniques. We used the results to investigate the relationship between immune population compositional alterations and disease stages and revealed a set of markers associated with multiple myeloma rapid progression.

## Materials and Methods

### Ethics Approval and Consent to Participate

All procedures performed in studies involving human participants were in accordance with the ethical standards of the Multiple Myeloma Research Foundation (MMRF) research committee. These samples provided by MMRF were all from the MMRF's CoMMpass clinical trial (NCT NCT01454297). Written informed patient consent was obtained from all patients for the collection and analysis of their samples by the MMRF. The CoMMpass study was conducted in accordance with recognized ethical guidelines in the United States and European Union. The Institutional Review Board at each participating center approved the study protocol.

### Ammonium-chloride-potassium Lysis of Bone Marrow Aspirates

Bone marrow aspirate (BMA) samples obtained from subjects enrolled in the MMRF CoMMpass study (NCT01454297). Any blood clots were removed from BMA samples via passage through 70 mmol/L cell strainer. BMA samples were aliquoted into 5 mL aliquots in 50 mL conical tubes and 45 mL of 22 mmol/L filtered ammonium-chloride-potassium (ACK) lysing buffer (155 mmol/L ammonium chloride/10 mmol/L potassium bicarbonate/0.1 mmol/L Ethylenediaminetetraacetic Acid (EDTA)/pH7.4) was added to each 5 mL aliquot and the tune gently inverted several times to mix. Tubes were then centrifuged at 400 × *g* for 5 minutes. The supernatant was removed and the cell pellet resuspended with 5 mL of RPMI1640 and transferred to a clean tube. All aliquots of ACK-lysed BMA aliquots were combined into 1 × 50 mL tube, the volume adjusted to 50 mL with RPMI1640. The cells were then mixed by gentle inversion and the tube centrifuged at 400 × *g* for 5 minutes. The supernatant was then removed by aspiration. Depending on the size of the BMA cell pellet, the cell pellet resuspended in 1–10 mL of EasySep buffer [PBS containing 2% FBS (v/v) and 1 mmol/L EDTA (PBS/FCS/EDTA buffer)]. A total of 25 mL of cell suspension was removed for cell counting.

### Isolation of CD138-positive and CD138-negative Cells from BMA

CD138-negative (CD138^−^) immune cell mononuclear cells in BMAs from subjects enrolled in the MMRF CoMMpass study (NCT NCT01454297) were isolated via negative selection from CD138-positive (CD138^+^) myeloma cells using the EasySep immunomagnetic bead technology (EasySep Human CD138-Positive Selection Kit: Stem Cell Technologies) in accordance with the manufacturers protocol. Briefly, 100 × 10^6^ cell/mL bone marrow mononuclear cell (BMMC) in a sterile 17 × 100 mm (14 mL) tube were gently mixed and incubated with 100 mL/mL CD138 selection antibody cocktail for 15 minutes at room temperature. A total of 50 mL/mL of EasySep magnetic nanoparticles was then added to the cell suspension, gently mixed, and incubated for a further 10 minutes at room temperature. The volume of the cell suspension was then adjusted to 8 mL with PBS containing 2% FBS (v/v) and 1 mmol/L EDTA (PBS/FCS/EDTA buffer) and the cell suspension mixed by gentle pipetting (2–3×). The tube was then placed in the magnetic separator. After 5 minutes incubation at room temperature, the magnet and tube were carefully inverted to pour off the supernatant into a sterile 50 mL conical tube. This supernatant contains the heterogeneous CD138^−^ immune cell mononuclear population (MNC). The tube was then removed from the magnet and an additional 8 mL of PBS/FCS/EDTA added, gently mixed, and returned to the magnetic separator. Again, after 5 minutes incubation in the magnetic separator, the tube and magnet were carefully inverted to pour of the supernatant into the 50 mL collection tube. This PBS/FCS/EDTA “wash” step was repeated once more resulting in approximately 24 mL suspension of CD138^−^ bone marrow MNCs. CD138^−^ MNCs were then pelleted by centrifugation at 400 × *g* for 5 minutes and the supernatant removed by aspiration. The CD138^−^ MNC pellet was resuspended in freezing medium (90% FCS/10% DMSO) at a concentration of approximately 8–10 × 10^6^ cells/mL prior to cryogenic storage in liquid nitrogen.

### Processing of BMMC and Library Prep From MMRF CoMMpass Study for scRNA-seq at Washington University in St. Louis

Washington University in St. Louis (WUSTL) Cell Thawing: Multiple myeloma BMMC aliquots were thawed in 37°C water bath. Cells were then pelleted by centrifugation at 300 × *g* for 5 minutes and all supernatant was removed. To prepare cells for the Miltenyi Dead Cell Removal Kit, cells were resuspended in 100 μL of beads and incubated at room temperature for 15 minutes. Dead cells were depleted using the autoMACSPro Separator. Live cells were pelleted by centrifugation at 450 × *g* for 5 minutes. Cells were finally resuspended in ice-cold PBS and 0.5% BSA and loaded onto the 10x Genomics Chromium Controller and using the Chromium Next GEM Single-Cell 3′ GEM, Library and Gel Bead Kit v3.3. Utilizing the 10x Genomics Chromium Single-Cell 3′v3 Library Kit and Chromium instrument, approximately 16,500 to 20,000 cells were partitioned into nanoliter droplets to achieve single-cell resolution for a maximum of 10,000 individual cells per sample. The resulting cDNA was tagged with a common 16nt cell barcode and 10nt Unique Molecular Identifier (UMI) during the Reverse Transcription (RT) reaction. Full-length cDNA from poly-A mRNA transcripts was enzymatically fragmented and size selected to optimize the cDNA amplicon size (∼400 bp) for library construction (10x Genomics). The concentration of the 10x single-cell library was accurately determined through qPCR (Kapa Biosystems) to produce cluster counts appropriate for the HiSeq 4000 or NovaSeq 6000 platform (Illumina). A total of 26 × 98 bp (3′v2 libraries) sequence data were generated targeting between 25K and 50K read pairs/cell, which provided digital gene expression profiles for each individual cell.

### Icahn School of Medicine at Mount Sinai BMMC Processing Differences From WUSTL

BMMC aliquots were partially thawed in 37°C water bath. A total of 1 mL of warm thawing media (RPMI + 10% FBS) was added to the partially thawed BMMC aliquot and the entire volume was transferred to a 15 mL conical tube containing 10 mL of warm thawing media. The empty BMMC tube was rinsed with another 1 mL of thawing media which was then also transferred to the 15 mL conical tube. Cells were processed using the EasySep Dead Cell Removal (Annexin V) Kit (StemCell Technologies, catalog no. 17899).

### scRNA-seq Data Quantification Preprocessing

For scRNA-seq analysis, the proprietary software tool Cell Ranger v3.0.0 from 10x Genomics was used for demultiplexing sequence data into FASTQ files, aligning reads to the human genome (GRCh38), and generating gene-by-cell UMI count matrix.

Seurat v3.0.0 (7, 8) was used for all subsequent analysis. First, a series of quality filters was applied to the data to remove those barcodes which fell into any one of these categories recommended by Seurat: too few total transcript counts (<300); possible debris with too few genes expressed (<200) and too few UMIs (<1,000); possible more than one cell with too many genes expressed (>50,000) and too many UMIs (>10,000); possible dead cell or a sign of cellular stress and apoptosis with too high proportion of mitochondrial gene expression over the total transcript counts (>20%). Finally, predicted doublets were also removed using scrublet V0.2.3.

We constructed a Seurat object using the unfiltered feature-barcode matrix for each sample. Each sample was scaled and normalized using Seurat's “SCTransform” function to correct for batch effects (with parameters: vars.to.regress = c("nCount_RNA", "percent.mito"), return.only.var.genes = F).

### scRNA-seq Cell Type Annotation

Cell types were assigned to each cluster by manually reviewing the expression of marker genes. The marker genes for main cell types were *CD79A*, *CD79B*, *MS4A1* (B cells); *CD8A*, *CD8B*, *CD7*, *CD3E* (CD8^+^ T cells); *CD4*, *IL7R*, *CD7*, *CD3E* (CD4^+^ T cells); *NKG7*, *GNLY, KLRD1, NCAM1* (NK cells); *MZB1*, *SDC1*, *IGHG1* (Plasma cells); *CLEC4C, IL3RA, IRF8*, GZMB (Dendritic cells); *FCGR3A* (Macrophages); *CD14*, *LYZ, S100A8, S100A9* (Monocytes); *AZU1, ELANE, MPO* (Neutrophils); *COL1A1, COL3A1, TNC, S100A4* (Fibroblasts); and *AHSP1*, *HBA*, *HBB* (Erythrocytes). Detailed cell type markers are listed in Supplementary Table S1A. All cells that were labeled as erythrocytes and plasma cells were removed from subsequent analysis.

### Processing of BMMC From MMRF CoMMpass Study for CITE-seq

Samples were thawed in the water bath at 37°C for 2–3 minutes and the cell concentration, viability were determined using a Bio-Rad T20 Cell Counter (catalog no. 145-0102). Samples were blocked by incubation with TruStain fcX (BioLegend, catalog no. 422301) in a 50 μL cell labeling buffer. Next, samples were labeled with Total-seq antibodies (BioLegend; Supplementary Table S1B) for 30 minutes. Cells were washed and resuspended to obtain a cell concentration of 700–1,200 cells/μL and gently pipette mix using a regular-bore pipette tip until a single-cell suspension is achieved. We then proceed immediately to Single-Cell Gene Expression Library (3′GEX) construction using 10X Chromium Single-Cell 3′ Reagent Kits v3 (catalog no. 1000075) and Chromium i7 Sample Index Plate with Barcoding technology for Cell Surface Protein. For each sample, 5,000 cells were injected for CITE-seq. The libraries were sequenced on NovaSeq S4 platform in pair end sequencing and a single index with at least 50,000 read pairs per cell.

### CITE-seq Data Quantification Preprocessing

We used Cell Ranger to demultiplex, map to the human reference genome (grch38), and count UMIs in the mRNA libraries, and CITE-seq-Count to count UMIs in the antibody-derived tag (ADT) libraries. We filtered out cells with more than 10% UMIs from mitochondrially encoded genes or less than 1,200 mRNA UMIs in total. We then constructed a Seurat object using the feature-barcode matrix for each sample (Seurat v3.0.0). Each sample was scaled and normalized using Seurat's “SCTransform” function to correct for batch effects (with parameters: vars.to.regress = c("nCount_RNA", "percent.mito"), return.only.var.genes = F). Next, the protein expression levels were added to the Seurat object, followed by normalization and scaling for ADT assay.

### CITE-seq Data Multimodal Integration and Cell Type Annotation

Using Citefuse v1.2.0, expression was normalized by function normaliseExprs(sce, altExp_name = "ADT", transform = "log"). We then integrated RNA and ADT matrix by an integration algorithm called similarity network fusion (SNF) and clustered cells by Louvain clustering. Then, cell types were assigned to each cluster by manually reviewing the expression of marker genes at RNA levels (same as scRNA-seq; Supplementary Table S1A) and ADT levels (if available). All cells that were labeled as erythrocytes and plasma cells were removed from subsequent analysis.

### Processing of BMMC From MMRF CoMMpass Study for CyTOF at Icahn School of Medicine at Mount Sinai

BMMC aliquots were thawed in a 37°C water bath and immediately transferred into RPMI + 10% FBS. Cells were pelleted by centrifugation at 300 × *g* for 5 minutes and all supernatant was removed. Cells were then incubated for 20 minutes in a 37°C water bath with Cell-ID Rh103 Intercalator (Fluidigm, catalog no. 201103A) to label nonviable cells. Samples were then blocked with Fc receptor blocking solution (BioLegend, catalog no. 422302) and stained with a cocktail of surface antibodies for 30 minutes on ice. All antibodies were either conjugated in-house using Fluidigm's × 8 polymer conjugation kits or purchased commercially from Fluidigm. Next, samples were fixed and barcoded using Fluidigm's 20-Plex Pd barcoding kit (catalog no. 201060) and pooled into a single tube. The pooled sample was then fixed and permeabilized using BD's Cytofix/Cytoperm Fixation/Permeabilization Kit (catalog no. 554714), blocked with heparin at a concentration of 100 U/mL to prevent nonspecific staining of eosinophils and stained with a cocktail of intracellular antibodies. Finally, the sample was refixed with freshly diluted 2.4% formaldehyde in PBS containing 0.02% saponin and Cell-ID Intercalator-Ir (Fluidigm, catalog no. 201192A) to label nucleated cells. The sample was then stored as a pellet in PBS until acquisition.

Immediately prior to acquisition, the pooled sample was washed with Cell Staining Buffer (CSB) and Cell Acquisition Solution (Fluidigm, catalog no. 201240) and resuspended in Cell Acquisition Solution at a concentration of 1 million cells per mL containing a 1:20 dilution of EQ normalization beads (Fluidigm, catalog no. 201078). The sample was acquired on the Fluidigm Helios mass cytometer using the wide bore injector configuration at an acquisition speed of < 400 cells per second.

### Processing of BMMC From MMRF CoMMpass Study for CyTOF at Mayo

BMMC aliquots were thawed in a 37°C water bath and immediately transferred into 15mL tubes and slowly diluted with 10 mL of prewarmed RPMI + 10% FBS+25 U/mL Benzonase (Sigma-Aldrich; catalog no. E1014-5KU; 250 U/mL). Cells were pelleted by centrifugation (all spins at 500 × *g* for 5 minutes) and supernatant was removed. Cells were then incubated for 1 hour in a 37°C water bath in 10 mL of RPMI+10% FBS. Cells were counted and 3–4 million cells were aliquoted into microfuge 2 mL conical tubes, pelleted and washed 2× with 2 mL CSB Maxpar Cell Staining Buffer (Fluidigm; catalog no. 201068; 500 mL) and resuspended in 300 μL of Cell-ID Cisplatin (Fluidigm; catalog no.: 201064) 5 minutes/RT, to label dead cells. Immediately quenched with 1.5 mL CSB, pelleted, and washed with CSB 2×.

For staining, the cell pellet was gently resuspended in 50 μL CSB and the addition of an equal volume of diluted surface antibody cocktail, for a final staining volume of 100 μL. The staining reaction was incubated on a rocker platform for 45 minutes at room temperature. A total of 1 mL of CSB was used to wash and pellet the cells 2×. Cell pellet was resuspended in the residual volume and then gently resuspended in 500 μL of 1× PBS. An equal volume of 4% PFA in PBS was added to fix cells for a minimum of 20 minutes at a final concentration of 2% PFA in PBS. The sample was labeled overnight at 4°C on a rocker platform with Cell-ID Intercalator-Ir (Fluidigm, catalog no. 201192A) in Maxpar Fix and Perm Buffer (Fluidigm; catalog no. 201067; 100 mL) to label nucleated cells.

The following day the sample was washed 1× with CSB (all cell pelleting performed at 800 × *g* for 5 minutes after fixation) and twice with Cell Acquisition Solution (Fluidigm, catalog no. 201240). Final resuspension was in Cell Acquisition Solution at a concentration of 0.7 million cells per mL containing a 1:10 dilution of EQ normalization beads (Fluidigm, catalog no. 201078). The sample was acquired on the Fluidigm Helios mass cytometer using the wide bore injector configuration at a targeted acquisition speed of 300 events per second. A cryopreserved specimen of 3–4 million Ficoll-enriched peripheral blood mononuclear cell (PBMC) derived from a pool of 4 anonymous platelet donors was included with every batch of MMRF samples (9). This sample was treated and analyzed in parallel throughout the entire experiment as a process control.

### Processing of BMMC From MMRF CoMMpass Study for CyTOF at Emory

BMMC aliquots were thawed in a 37°C water bath and immediately transferred into RPMI+10% FBS. Cells were pelleted by centrifugation at 300 × *g* for 5 minutes and all supernatant was removed. Cells were then incubated for 20 minutes in a 37°C incubator. Cells were pelleted by centrifugation at 300 × *g* for 5 minutes and all supernatant was removed. Cells were resuspended in PBS and incubated with cisplatin for 1 minute (Fluidigm, catalog no. 201195) to label nonviable cells. Samples were washed with Maxpar cell staining buffer (Fluidigm, catalog no. 201068) and stained with a cocktail of surface antibodies for 15 minutes at room temperature. All antibodies were either conjugated in-house using Fluidigm's X8 polymer conjugation kits or purchased commercially from Fluidigm. Next, samples were washed and fixed and permed with TF Fix/Perm and Perm/Wash Kit (BD Pharmigen, catalog nos. 51-9008100 and 51-9008102) using manufacturer's recommendations. Permeabilized samples were incubated for 30 minutes in Perm/Wash with a cocktail of intracellular antibodies. After washing and centrifugation at 800 × *g* for 5 minutes, the sample was refixed with Maxpar Fix I buffer (Fluidigm, catalog no. 201065) and Cell-ID Intercalator-Ir (Fluidigm, catalog no. 201192A) to label nucleated cells. The sample was then stored as a pellet in PBS until acquisition. Immediately prior to acquisition, the sample was washed with Cell Staining Buffer and Maxpar Water (Fluidigm, catalog no. 201069) and resuspended in Maxpar Water at a concentration of 1 million cells per mL containing a 1:10 dilution of EQ normalization beads (Fluidigm, catalog no. 201078). The sample was acquired on the Fluidigm Helios mass cytometer using the HT injector configuration at an acquisition speed of <500 cells per second.

### CyTOF Data Preprocessing

The resulting FCS files were normalized and concatenated using Fluidigm's CyTOF software and then demultiplexed using the Zunder lab single-cell debarocder (https://github.com/zunderlab/single-cell-debarcoder). The FCS files were further cleaned on Cytobank by removing EQ beads, low DNA debris, and gaussian multiplets. Barcoding multiplets were also removed on the basis of the Mahalanobis distance and barcode separation distance parameters provided by the Zunder lab debarcoder.

### CyTOF Cell Type Annotation and Expression Normalization

Gating and data analysis were done using WUSTL Cytobank. Live, single cells are selected by gating out cells/debris with outlier cisplatin and DNA intercalator staining. Cell populations were determined on the basis of gating of cell type marker expression. Icahn School of Medicine at Mount Sinai (**ISMMS)**: CD3^+^CD19^−^CD56^−^CD33^−^ (T cells); CD3^−^CD19^−^CD56^−^CD33^−^CD123^+^HLA_DR+CD11c^+^ [plasmacytoid dendritic cells (pDC)]; CD3^−^CD19^+^CD56^−^CD33^−^ (B cells); CD56^+^CD3^−^CD19^−^CD33^−^ (NK cells); CD33^+^CD3^−^CD19^−^CD14^+^ (monocytes); CD33^+^CD3^−^CD19^−^CD14^−^CD16^+^ (macrophages). **Mayo:** CD3^+^CD19^−^ (T cells); CD3^−^ CD19^+^CD56^−^ (B cells); CD56^+^CD3^−^CD16^+^HLADR−/CD56^+^CD3^−^CD16^−^CD123^−^CD11c^−^ (NK cells); CD3^−^CD19^−^CD20^−^CD14^+^ (monocytes); CD3^−^CD19^−^CD20^−^CD14^−^CD16^+^ (macrophages); CD3^−^CD19^−^CD20^−^CD123^+^ (pDC). **Emory**: CD3^+^CD19^−^ (T cells); CD3^−^ CD19^+^ (B cells); CD3^−^CD19^−^CD14^+^ (monocytes); CD3^−^CD19^−^CD14^−^CD16^+^ (macrophages). For T-cell subtypes, ISMMS and Mayo used the same gating strategy: CD4^+^CD8^−^ (CD4^+^ T cells); CD8^+^CD4^−^ (CD8^+^ T cells); CD4^+^CD8^−^CD25^+^CD127^−^ [regulatory T cell (Treg)]; CD45RA^+^CCR7^+^ (naïve T cells); CD45RA^+^CCR7^−^ (EMRA T cells); CD45RA^−^CCR7^+^ (central memory T cells), CD45RA^−^CCR7^−^ (effector memory T cells), Emory: CD4^+^CD8^−^ (CD4^+^ T cells); CD8^+^CD4^−^ (CD8^+^ T cells); CD45RO^−^CCR7^+^ (naïve T cells). Next, we performed t-SNE analysis for 18 samples from ISMMS. We used the scaled expression of markers, including CD57, CD11c, Ki67, CD19, CD45RA, KLRG1, CD4, CD8, ICOS, CD16, CD127, CD1c, CD123, CD66b, TIGIT, TIM3, CD27, PD-L1, CD33, CD14, CD56, NKG2A, CD5, CD45RO, NKG2D, CD25, CCR7, CD3, Tbet, CD38, CD39, CD28, DNAM1, HLA-DR, PD-1, Granzyme B, CD11b. For expression normalization in CyTOF analysis, we followed instructions from Cytobank and used transformed ratios itself compared with its control, which is the table's minimum of median of channel (described here https://support.cytobank.org/hc/en-us/articles/206147637-How-to-create-and-configure-a-Heatmap).

### Bland-Altman Analysis

R package Blandr (v0.5.3) was used to calculate mean difference and 95% confidence interval (CI) in Bland-Altman analyses (10). Parameter sig.level = 0.95.

### Differential Expression Analysis

Differential expression analysis was performed using the default test (Wilcoxon rank-sum test) of function FindMarkers (from the Seurat package) with the specified parameters: min.pct = 0.25, logfc.threshold = 0.25, and only.pos = T.

### Data Availability Statement

The sequence data generated in this study have been submitted to the NCBI BioProject database PRJNA765009 (https://www.ncbi.nlm.nih.gov/bioproject/).

## Results

### Patient Characteristics and Overview of CD45^+^ Immune Cells Measured by scRNA-seq, CyTOF, and CITE-seq

**FIGURE 1:**
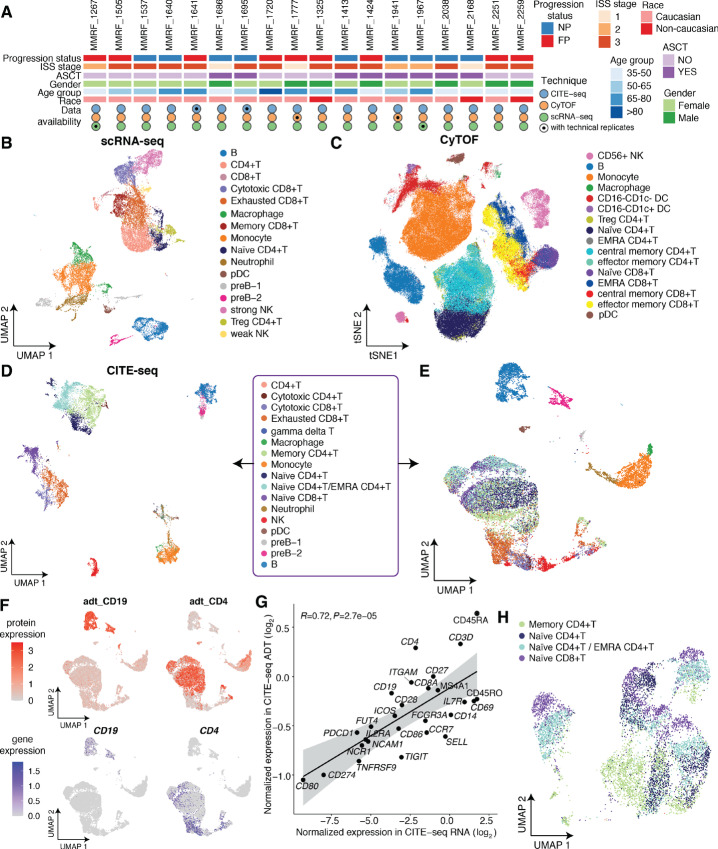
Overview of cell populations of 18 multiple myeloma patient samples subject to scRNA-seq, CyTOF, and CITE-seq. **A,** Patient characteristics and single-cell data collection. FP and NP denote fast progressors and nonprogressors, respectively. ISS = International Staging System. ASCT = Autologous Stem Cell Transplantation. **B,** UMAP projection of integrated scRNA-seq data, with cells colored by immune cell types. **C,** t-SNE projection of integrated CyTOF data, with cells colored by immune cell types. **D,** UMAP projection of integrated CITE-seq data, with cells clustered by integrated RNA and ADT expression, colored by immune cell types. **E,** UMAP projection of integrated CITE-seq data, with cells clustered by transcriptional level alone, colored by immune cell identities from **D**. **F,** Comparison of canonical cell type marker gene expressions between protein level (ADT, top) and transcriptional level (RNA, bottom). Cells are colored by normalized expression. **G,** Concordance of sample-level average expressions of CITE-seq protein markers measured at RNA level and ADT level. The gray shaded area represents the 95% confidence interval around the line of best fit. *R* = Pearson correlation coefficient. **H,** UMAP projection of CD4^+^ T cells and naïve CD8^+^ T cells, which is the subset of integrated data in **E**, with cells clustered by transcriptional level alone, colored by immune cell identities from **D** and **E**.

We used 18 cryopreserved multiple myeloma samples of CD138^−^ “immune cell” fractions from patients enrolled in the MMRF CoMMpass study (NCT01454297). Nine were fast progressors (FP, progressed within 6 months) and nine were nonprogressors (NP, progressed >6 months but within 5 years) with patient ages ranging from 37 to 83 years. Twelve patients were in the ISS stage III, 8 underwent autologous stem cell transplantation (ASCT), 11 were females and 15 were Caucasians (Fig. 1A; Supplementary Table S1C). Each sample was subjected to scRNA-seq, CyTOF, and CITE-seq at three different respective academic research centers, namely WUSTL, ISMMS, and Beth Israel Deaconess Medical Center (BIDMC). All sites received aliquots from the same sample and technical replicates were conducted for two samples for each assay (Fig. 1A).

To assess immune cell composition of patients with multiple myeloma, bone marrow (BM) baseline samples (collected at the initial diagnosis) from these 18 patients were subjected to scRNA-seq, with immune cells clustered on the basis of their transcriptome profiles using the Louvain clustering algorithm implemented by Seurat (refs. 7, 8; Fig. 1B). We then investigated immune cells of these same samples by CyTOF using a 39-marker panel (Supplementary Table S1D). Cell populations were characterized by expression of markers, clustered by the flowsom algorithm (11), and visualized with vi-SNE in the Cytobank (12) platform (Fig. 1C). Given the discordance between RNA expression and protein expression that is known to exist (13), it is informative to characterize cell populations by measuring RNA and protein at the same time. Finally, we utilized CITE-seq with antibody-oligonucleotide conjugates and 29 protein markers (Supplementary Table S1B) to simultaneously quantify single-cell transcriptomes and surface proteins. Following standard scRNA-seq quality filtering protocols, immune cells were clustered on the basis of integrated multi-omic profiles by the SNF integration algorithm in CiteFuse (ref. 14; Fig. 1D). From CD138^−^ BM aliquots, we detected, on average, 1,051 immune cells/sample using scRNA-seq, >64K CD45^+^ cells/sample using CyTOF, and 718 immune cells/sample using CITE-seq.

### Advantages of CITE-seq in Distinguishing T-cell Subtypes in Multiple Myeloma

To assess the potential advantages of simultaneous quantification of RNA and protein expression in CITE-seq as compared with standard scRNA-seq, we labeled immune cell identities determined by integrated transcriptome and protein expression, but clustered cells by transcriptional profiles alone (Fig. 1E). Interestingly, most cell types, including B cells, monocytes, macrophages, neutrophils, and pDCs, formed distinct clusters, while T-cell subtypes mixed together. To further understand the difference of cell type marker expression between the RNA and protein levels, we visualized the expression of some canonical markers in Uniform Manifold Approximation and Projection (UMAP) and investigated the concordance of the sample-level average expression of the 29 CITE-seq protein markers between RNA level and ADT level (Fig. 1F and G; Supplementary Fig. S1A). As expected, expression levels of markers are generally concordant (*R* = 0.72, *P* < 10^−4^), with some exceptions where protein-level expression is higher than RNA-level expression and vice versa. One impressive example is CD4 (Fig. 1F and G), which is highly expressed at ADT measurement, but minimally expressed at the RNA level, mainly because mRNAs are produced at much lower rates and have much shorter half-lives than proteins (15). This observation is consistent with previous studies showing low CD4 mRNA expression compared with surface CD4 protein (16). Finally, because naïve CD8^+^ T cells were clustered together with CD4^+^ T cells based on transcriptome profiles (Fig. 1E), we investigated whether reclustering T cells alone could help to distinguish subtypes at the RNA level. Because the high similarities of transcriptional profiles among T cells (16) and different surface protein markers could be encoded by the same gene (17), reclustering CD4^+^ and naïve CD8^+^ T cells did not provide additional resolution of T-cell subtypes (Fig. 1H). Consistent with a published study about renal T subtype identification using CITE-seq (18), our observation emphasizes the advantage of integrating protein-level expression of cell type markers for multiple myeloma T-cell subtype identification in CITE-seq as compared with standard scRNA-seq.

### Data Reproducibility and Comparisons of Cell Populations Measured by the Same Technologies Across Different Centers

To examine data reproducibility, percentages of cell subsets in CD45^+^ populations were compared between technical replicates for two samples in each assay. The technical replicate pairs are strongly correlated in all three assays (average Pearson correlation coefficient *r* = 0.94 in scRNA-seq, 0.89 in CyTOF, and 0.92 in CITE-seq; Supplementary Fig. S1B–S1D). Next, to examine the consistency of immune cell populations measured by the same techniques at different sites, we evaluated the percentage of immune populations captured by three centers using four samples. scRNA-seq data were generated in ISMMS, WUSTL, and BIDMC using aliquots of the same samples and CyTOF data were generated in ISMMS, Mayo Clinic, and Emory University (panels are shown in Supplementary Table S1D–S1F). BIDMC scRNA-seq data are from CITE-seq data analyzed with RNA signal alone (Supplementary Fig. S1E). We observed that the percentages of B cells, pre-B cells, NK cells, pDCs, monocytes and macrophages are generally consistent, while the T-cell subset varies across centers in scRNA-seq measurement (Supplementary Fig. S1F). This suggests that T-cell composition could vary by aliquots and potential sample processing differences across centers while other cell types are more similar in scRNA-seq measurement. The cell type abundance measured by CyTOF is less variable than that measured by scRNA-seq, with smaller differences observed in T-cell subsets across centers (Supplementary Fig. S1G, mean difference calculated by Bland-Altman analysis, shown in Supplementary Table S1G). Moreover, cell subset abundances of ISMMS samples tend to have less variation likely due to the benefit of barcoding samples (Materials and Methods). The cell type frequencies calculated by one center (Emory) tend to be lower overall compared with other centers in CyTOF, probably because wide bore injector assembly with cell acquisition solution was not used to maintain cell integrity (Materials and Methods). It is worthwhile noting that including reference samples in CyTOF is very helpful for identifying potential artifacts. For example, we observed a big proportion of CD66b/CD3^+^ cells in patient samples while these were absent in the reference sample from a healthy donor (data not shown). We hypothesized that this CD66b staining artifact (CD66b is not expressed on CD3^+^ T cells) was likely due to nonspecific staining from dead cells. Indeed, the percentage of CD66b/CD3^+^ cells dropped dramatically after dead cell depletion. Finally, to evaluate the similarity of expression profiles across different samples and centers, we calculated the Pearson correlation coefficient of expression of the B-cell markers between populations detected from different centers using scRNA-seq (Supplementary Fig. S1H). We observed that B cells clustered according to patients instead of centers, suggesting patient dependence of B-cell transcriptome profiles, likely because B cells are potential reservoirs of plasma cells (19). Overall, we observed that cell type abundances are generally consistent across centers for most cell types and that similarity of transcriptome profiles of immune populations is center independent, suggesting absence of strong batch effects across centers. These observations imply that our cross-technique comparisons should be valid.

### Comparisons of Cell Type Abundances and Correlations of Cell Type Marker Expression Across the Three Techniques

**FIGURE 2:**
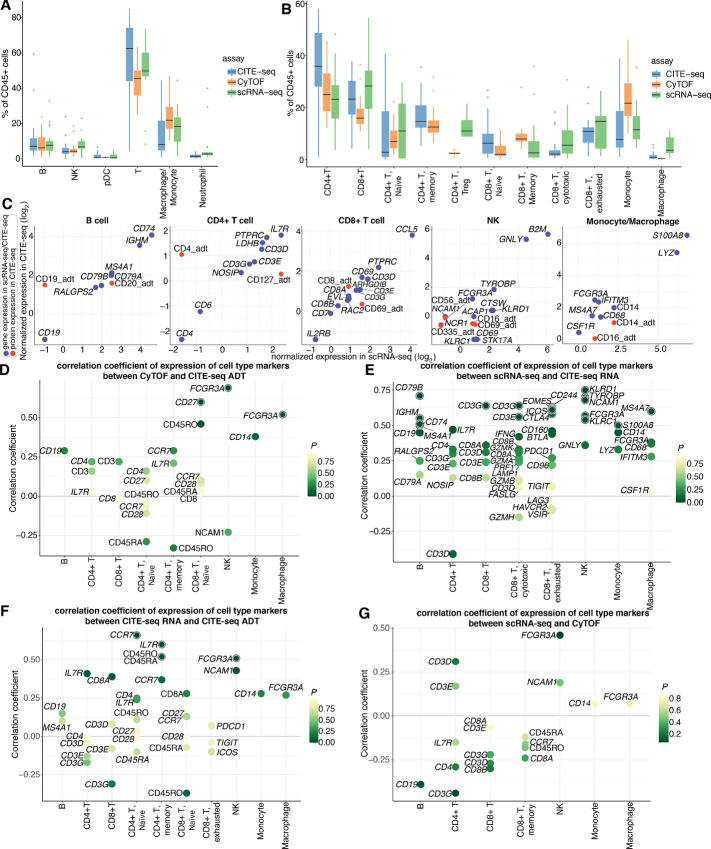
Comparison of cell subset frequencies and correlations of expression of canonical cell type markers across different modalities. **A,** Main immune cell population (CD45^+^) frequencies observed by CITE-seq, CyTOF, and scRNA-seq. Each boxplot is colored by assay. CITE-seq populations are determined on the basis of integrated RNA and ADT expressions. **B,** Immune cell subtype frequencies for CITE-seq, CyTOF, and scRNA-seq. Each boxplot is colored by assay. CITE-seq populations are determined on the basis of integrated RNA and ADT expressions. **C,** Concordance of sample-level average expressions of canonical cell type markers in main cell subsets between scRNA-seq and CITE-seq. CITE-seq RNA and protein (ADT) level expressions are represented by blue and red dots, respectively. **D,** Spearman correlation coefficients of protein level expressions of cell type markers between CyTOF and CITE-seq. Each dot represents a marker gene and the color of the dot represents the *P* value of correlation. Markers are highlighted with an outer circle if the *P* value is less than 0.05. **E,** Spearman correlation coefficients of transcriptional level expressions of cell type markers between scRNA-seq and CITE-seq. Each dot represents a marker gene and the color of the dot represents the *p* value of correlation. Markers are highlighted with an outer circle if the *P* value is less than 0.05. **F,** Spearman correlation coefficients of cell type markers between transcriptional level and protein level expressions in CITE-seq. Each dot represents a marker gene and the color of the dot represents the *P* value of correlation. Markers are highlighted with an outer circle if the *P* value is less than 0.05. **G,** Spearman correlation coefficients of cell type markers between transcriptional level expressions from scRNA-seq and protein level expressions from CyTOF. Each dot represents a marker gene and the color of the dot represents the *P* value of correlation. Markers are highlighted with an outer circle if the *P* value is less than 0.05.

To evaluate the concordance of cell type composition determined by the three methods, we calculated the cell subset frequency of each immune population relative to the CD45^+^ populations (Fig. 2A). Overall, all three approaches were concordant, though there is somewhat stronger concordance between scRNA-seq and CITE-seq for all cell types except NK cells (mean difference calculated by Bland-Altman analysis, shown in Supplementary Table S1H). Cell type abundance is especially consistent for B cells, pDC, and neutrophils. Interestingly, the cell frequency decreased and increased for T cells and macrophages/monocytes, respectively, in CyTOF as compared with scRNA-seq and CITE-seq. The mean differences between CyTOF and CITE-seq were −13.6% (95% CI: −24.02 to −3.11) for T cells and 11.07% (95% CI: 3.19–18.95) for macrophages/monocytes. This finding is consistent with a previous study where fewer T cells were detected in CyTOF compared with scRNA-seq in healthy bone marrow samples (20). To further investigate which subpopulations were discordant, the frequencies of T-cell subsets, monocytes, and macrophages were evaluated (Fig. 2B, mean difference calculated by Bland-Altman analysis). Interestingly, CITE-seq detected far more CD4^+^ T cells compared with CyTOF and scRNA-seq, while CyTOF detected far fewer CD8^+^ T cells compared with the other two techniques. In terms of T-cell subtypes, Treg frequency increased and memory CD8^+^ T cells reduced in scRNA-seq, as compared with CyTOF. In addition, scRNA-seq detected far more macrophages than the other two methods, while monocyte frequency was the lowest in CyTOF.

To further evaluate concordance between scRNA-seq and CITE-seq, we examined expression of cell type marker genes, including both the RNA and ADT levels. Average expressions of each marker gene at the transcriptional level (blue dots) between scRNA-seq and CITE-seq are generally concordant (Fig. 2C). In contrast, we observed drastic differences of some marker genes between RNA and ADT expression in CITE-seq, probably due to the RNA dropout (21) and shorter half-lives of mRNAs versus proteins (15). For example, expression of CD4_adt is higher than that of transcriptional CD4, whereas CD127/*IL7R* tends to be highly expressed at the transcriptional level. This dynamic explains why *IL7R* is often differentially expressed in CD4^+^ T-cell population, while CD4 is weakly expressed in scRNA-seq. Taken together, these observations highlight the importance of choosing cell type marker genes best suited to particular modalities.

We also correlated expressions of marker genes among scRNA-seq, CyTOF, and CITE-seq. The vast majority are positively correlated in protein–protein comparison (Fig. 2D) and RNA–RNA comparison (Fig. 2E). Next, we investigated the correlations of expressions of marker genes between the transcriptome and protein levels (Fig. 2F and G; Supplementary Fig. S2A and S2B). As expected, the overall correlation between different modalities is lower than that of the same modalities. We observed significant correlation for some markers, including *CCR7* in CD4^+^ naïve T cells, *IL7R* in CD4^+^ memory T cells, and *FCGR3A* in NK cells, between RNA and protein level of CITE-seq, while no markers are significantly correlated between scRNA-seq and CyTOF (Fig. 2G). We also found that *FCGR3A* in macrophages has a strong correlation, while some markers are significantly anticorrelated between CITE-seq transcriptional level and CyTOF, such as *CD3D*, *CD3G*, *IL7R*, *CD8A*, etc. (Supplementary Fig. S2A–S2C; Supplementary Table S1I).

### Decreased Ratio of CD4^+^/CD8^+^ T Cells From ISS Stage 2 to ISS Stage 3 Patients and FP-related Gene Signatures

**FIGURE 3:**
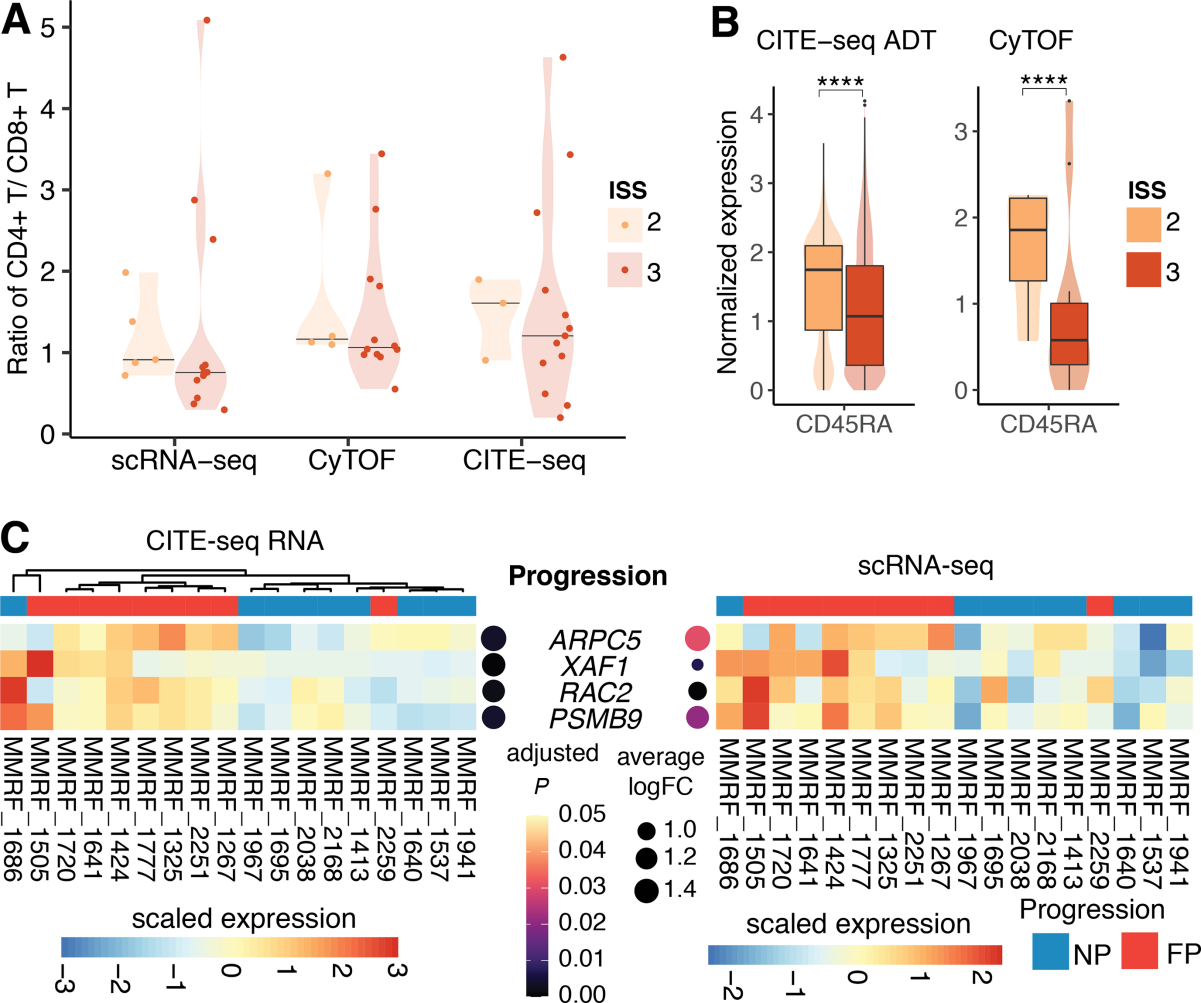
Ratio of CD4^+^ T/CD8^+^ T of patients in different ISS stages and markers associated with ISS disease stages and multiple myeloma progression. **A,** Violin plots showing the ratio of CD4^+^ T/CD8^+^ T of patients in ISS stage 2 and 3 in scRNA-seq, CyTOF, and CITE-seq. Horizontal lines indicate the median of data points in each group. **B,** Violin plots showing single cell–level normalized expression of CD45RA in CITE-seq ADT measurement and CyTOF. The difference is significant at *P* ≤ 0.0001 based on Wilcoxon rank-sum test. **C,** Heatmaps showing DEGs of NK cells of FP versus NP patients in CITE-seq RNA measurement (left) and scRNA-seq measurement (right). The samples are ordered on the basis of hierarchical clustering of expression profiles of these genes in CITE-seq RNA measurement. Expression values are scaled such that for each gene, the average of the scaled expression is 0 and the SD is 1. Adjusted *P* values and log fold change in CITE-seq and scRNA-seq were shown on the left and right side of DEGs, respectively. FC = fold change.

Furthermore, we sought to investigate the relationship between clinical features and immune cell composition of patients with multiple myeloma by examining the ratio of CD4^+^/CD8^+^ T cells of patients at different disease stages. A previous study used flow cytometry to reveal that this ratio was significantly lower in PBMCs of patients with multiple myeloma as compared with that of normal controls and the ratio decreased with the multiple myeloma progression (5). By integrating three assays, we found the ratio tends to decrease from ISS stage 2 to ISS stage 3 patients (Fig. 3A). Furthermore, CITE-seq and CyTOF analyses revealed significant downregulation of CD45RA in stage 3 patients, suggesting that CD8^+^ T cells tend to be activated rather than naïve in stage 3 patients (Fig. 3B). In addition, we then identified several differentially expressed genes (DEG) of NK cells from FPs relative to NPs, including *ARPC5*, *XAF1*, *RAC2*, and *PSMB9*, as revealed by both scRNA-seq and CITE-seq assays (Fig. 3C). *ARPC5*, actin-related protein 2/3 complex subunit 5, has been revealed to be highly expressed in patients with poor overall survival and could be treated as an independent biomarker for patients with multiple myeloma (22), consistent with our observations. A previous microarray-based study found that *RAC2*, Rac family small GTPase 2, is significantly upregulated in multiple myeloma as compared with MGUS (23). One subunit of the proteasome (PSMB9), was remarkably highly expressed in cell groups with t(4;14) translocations versus cells from MGUS (24). In summary, previous studies indicated *RAC2* and *PSMB9* are associated with disease development from MGUS to multiple myeloma and our analysis suggested that they might also be related to multiple myeloma progression. Taken together, we observed the ratio of CD4^+^ T/CD8^+^ T cells decreased in stage 3 patients relative to stage 2 patients, suggesting an increased population of CD8^+^ T cells in bone marrow microenvironment (BMME) of patients in stage 3. We also found that *RAC2* and *PSMB9* are upregulated in NK cells in FPs relative to NPs at transcriptional level, which could potentially serve as multiple myeloma progression markers.

## Discussion

Single-cell sequencing technologies have been widely used in studying tissue heterogeneity, tumorigenesis, and metastasis given their advantages of being able to depict genome, transcriptome, proteome, and other mutli-omics profiles of single cells (25). However, the similarities of measurements across the various single-cell techniques remain to be fully elucidated. Herein, we integrated scRNA-seq, CyTOF, and CITE-seq to perform a detailed comparison of their measurements for multiple myeloma BMME. From CD138^−^ BM aliquots of 20 samples from 18 patients, we detected, on average, 1,051 immune cells/sample using scRNA-seq, >64K CD45^+^ cells/sample using CyTOF, and 718 immune cells/sample using CITE-seq. By clustering cells with or without protein profiles in CITE-seq, we showed the advantages of multimodal measurement over transcriptional measurement alone of cell type markers when characterizing T-cell subtypes in MM (Fig. 1E–H). This observation is in line with a study to investigate renal T-cell subtypes by CITE-seq (18).

Next, to examine the consistency of cell populations measured by the same techniques at different sites, we evaluated the cell subset abundances captured by three centers using four samples. Cross-center comparisons (Supplementary Fig. S1F and S1G) suggested no strong batch effect across centers and there are some important factors to consider to obtain reproducible and reliable results: (i) It is important to include reference samples in CyTOF to help identify marker nonspecific staining artifacts; (ii) Barcoding samples, sample delivery mechanism, and using lyophilized panels is important in CyTOF experiments. Furthermore, cross-technique comparisons revealed that the percentages of immune populations measured by scRNA-seq, CyTOF, and CITE-seq are generally concordant, except some variations in T cells, macrophages, and monocytes (Fig. 2A and B). Analysis revealed relatively high correlations of most markers between the same modalities, though some markers are negatively correlated. (Fig. 2C–G). This observation highlighted the importance of choosing marker genes best suited to particular modalities.

Previous studies have found patients with multiple myeloma have lower CD4^+^ T/CD8^+^ T ratios relative to healthy donors and these ratios are further decreased in ISS stage 3 versus ISS stage 1 patients (5). Here, we confirmed this trend using three single-cell technologies, finding that this ratio tends to decrease even in stage 3 versus stage 2 patients (Fig. 3A). We also observed the decreased ratio in stage 2 compared with stage 1 patients based on CyTOF and CITE-seq measurement but not in scRNA-seq, probably due to the limited number of patients in stage 1. Future study could further investigate how immune cell composition changes along with ISS stages with expanded sample size. In addition, we observed upregulation of *ARPC5*, *XAF1*, *RAC2*, and *PSMB9* in NK cells of FPs compared with those of NPs, as suggested by both scRNA-seq and CITE-seq RNA measurements (Fig. 3C). *RAC2* and *PSMB9* have been revealed to be associated with disease development from MGUS to multiple myeloma (23, 24) and our analysis suggested that they might also be related to multiple myeloma rapid progression, supported by both scRNA-seq and CITE-seq. Because of the limited number of protein markers in CITE-seq, we were unable to evaluate the protein-level expression of these multiple myeloma progression-related genes identified from RNA measurement, which requires further validation. It would also be interesting to investigate multiple myeloma progression-related markers after controlling for treatments in future studies.

This analysis is just a small sampling of the larger work being conducted by the MMRF and their associated academic research centers to provide a sufficiently broad, deep, and technologically diverse vast dataset for accurately characterizing BMME and to help interrogate multiple myeloma TME using different single-cell technologies. We hope this study will help researchers refine cell population characterization strategies and provide insights to those considering integrating multiple single-cell methods to comprehensively address biological questions.

## Supplementary Material

Supplementary Figure FS1 Expression of cell type markers in CITE-seq and comparison of cell subset abundance between technical replicates and across different centers

Supplementary Figure FS2 Correlation of expression of canonical cell type markers across different modalities

Supplementary Table TS1 supplementary table 1 shows cell type annotation markers, clinical information of patients, cross-technique comparison of cell subset abundance and expression of cell type marker genes in CITE-seq and CyTOF
