# Supplementary material for: Comprehensive Characterization of the Multiple Myeloma Immune Microenvironment Using Integrated scRNA-seq, CyTOF, and CITE-seq Analysis
Source: Cancer Res Commun. 2022 Oct 25;2(10):1255–65. doi: 10.1158/2767-9764.CRC-22-0022 (PMC10035369; doi:10.1158/2767-9764.CRC-22-0022)
Supplement: Supplementary Figure FS1 — Expression of cell type markers in CITE-seq and comparison of cell subset abundance between technical replicates and across different centers [file crc-22-0022-s01.pdf]

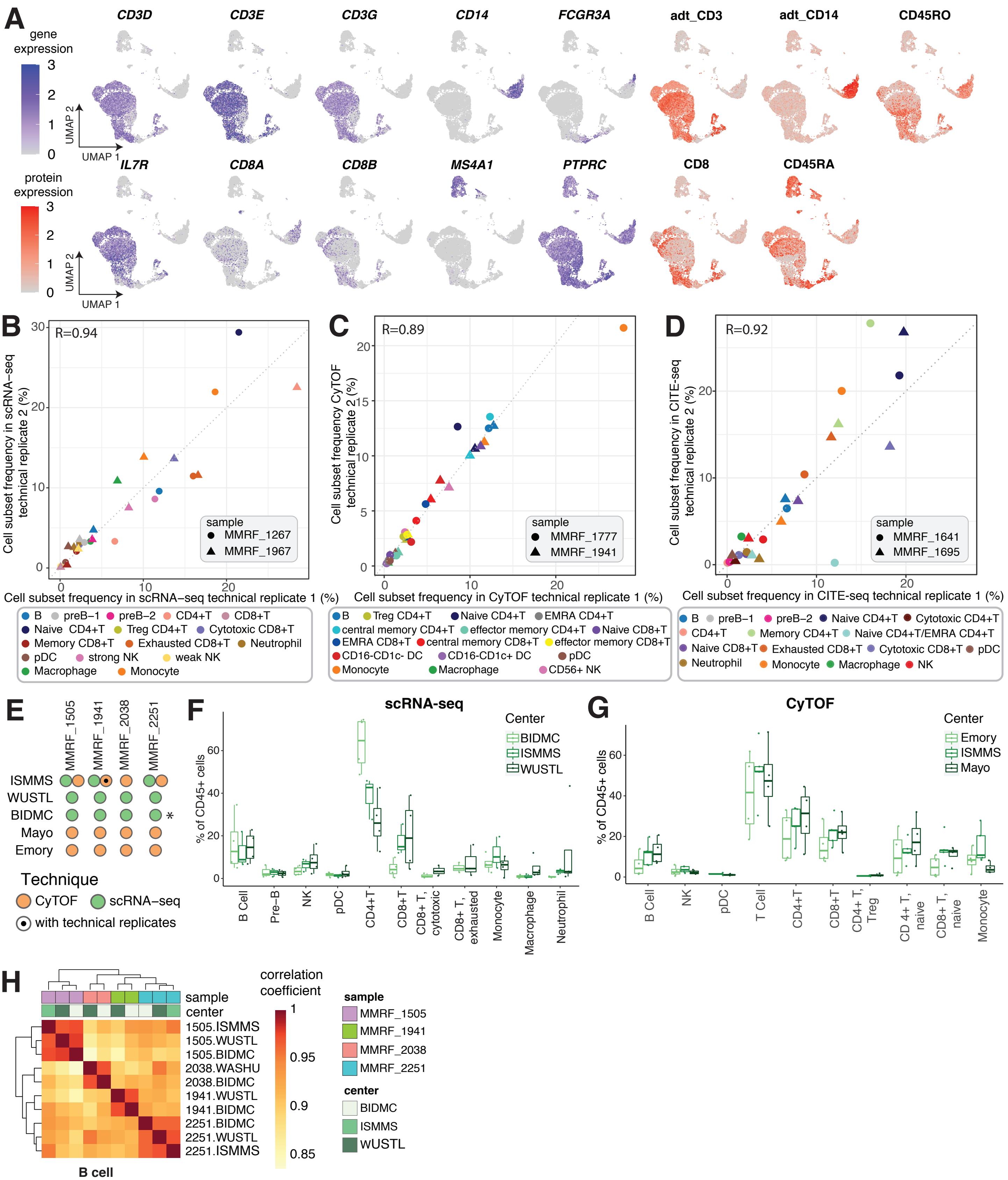
A
gene expression
CD3D
CD3E
CD3G
CD14
FCGR3A
adt_CD3
adt_CD14
CD45RO
IL7R
CD8A
CD8B
MS4A1
PTPRC
CD8
CD45RA
protein expression
UMAP 1
UMAP 2
B
R=0.94
Cell subset frequency in scRNA-seq technical replicate 2 (%)
Cell subset frequency in scRNA-seq technical replicate 1 (%)
sample
MMRF_1267
MMRF_1967
C
R=0.89
Cell subset frequency CyTOF technical replicate 2 (%)
Cell subset frequency in CyTOF technical replicate 1 (%)
sample
MMRF_1777
MMRF_1941
D
R=0.92
Cell subset frequency in CITE-seq technical replicate 2 (%)
Cell subset frequency in CITE-seq technical replicate 1 (%)
sample
MMRF_1641
MMRF_1695
E
MMRF_1505
MMRF_1941
MMRF_2038
MMRF_2251
ISMMS
WUSTL
BIDMC
Mayo
Emory
Technique
CyTOF
scRNA-seq
with technical replicates
F
scRNA-seq
% of CD45+ cells
Center
BIDMC
ISMMS
WUSTL
G
CyTOF
Center
Emory
ISMMS
Mayo
H
sample
center
correlation coefficient
B cell
FIGURE S1

Fig. S1

Expression of cell type markers in CITE-seq and comparison of cell subset abundance between technical replicates and across different centers.

A) Gene and protein expressions of canonical cell type markers for CITE-seq (related to Fig. 1F). Cells are colored by normalized expression.

B) Reproducibility of technical replicates for scRNA-seq in terms of cell subset frequency. Linear regression line is shown in grey. Dots are colored by immune cell types and shaped by samples. R = Pearson correlation coefficient.

C) Reproducibility of technical replicates for CyTOF. Dots are colored by immune cell types and shaped by samples. R = Pearson correlation coefficient.

D) Reproducibility of technical replicates for CITE-seq. CITE-seq populations are determined by integrated RNA and ADT expressions. Dots are colored by immune cell types and shaped by samples. R = Pearson correlation coefficient.

E) Overview of datasets used in cross-center comparisons. * denotes that BIDMC scRNA-seq data is from CITE-seq with cells analyzed using RNA signal alone.

F) Immune cell population (CD45+) frequencies observed by scRNA-seq in three different centers. Boxplot is colored by center.

G) Immune cell population (CD45+) frequencies observed by CyTOF in three different centers. Boxplot is colored by center.

H) A heatmap showing Pearson correlation coefficient of expression profiles of B cell marker genes in B cells between populations detected from different centers using scRNA-seq. B cell markers include *CD79A, CD79B, MS4A1* and pre-B cell markers listed in the Supplementary Table S1B.
