# Supplementary material for: Comprehensive Characterization of the Multiple Myeloma Immune Microenvironment Using Integrated scRNA-seq, CyTOF, and CITE-seq Analysis
Source: Cancer Res Commun. 2022 Oct 25;2(10):1255–65. doi: 10.1158/2767-9764.CRC-22-0022 (PMC10035369; doi:10.1158/2767-9764.CRC-22-0022)
Supplement: Supplementary Figure FS2 — Correlation of expression of canonical cell type markers across different modalities [file crc-22-0022-s02.pdf]

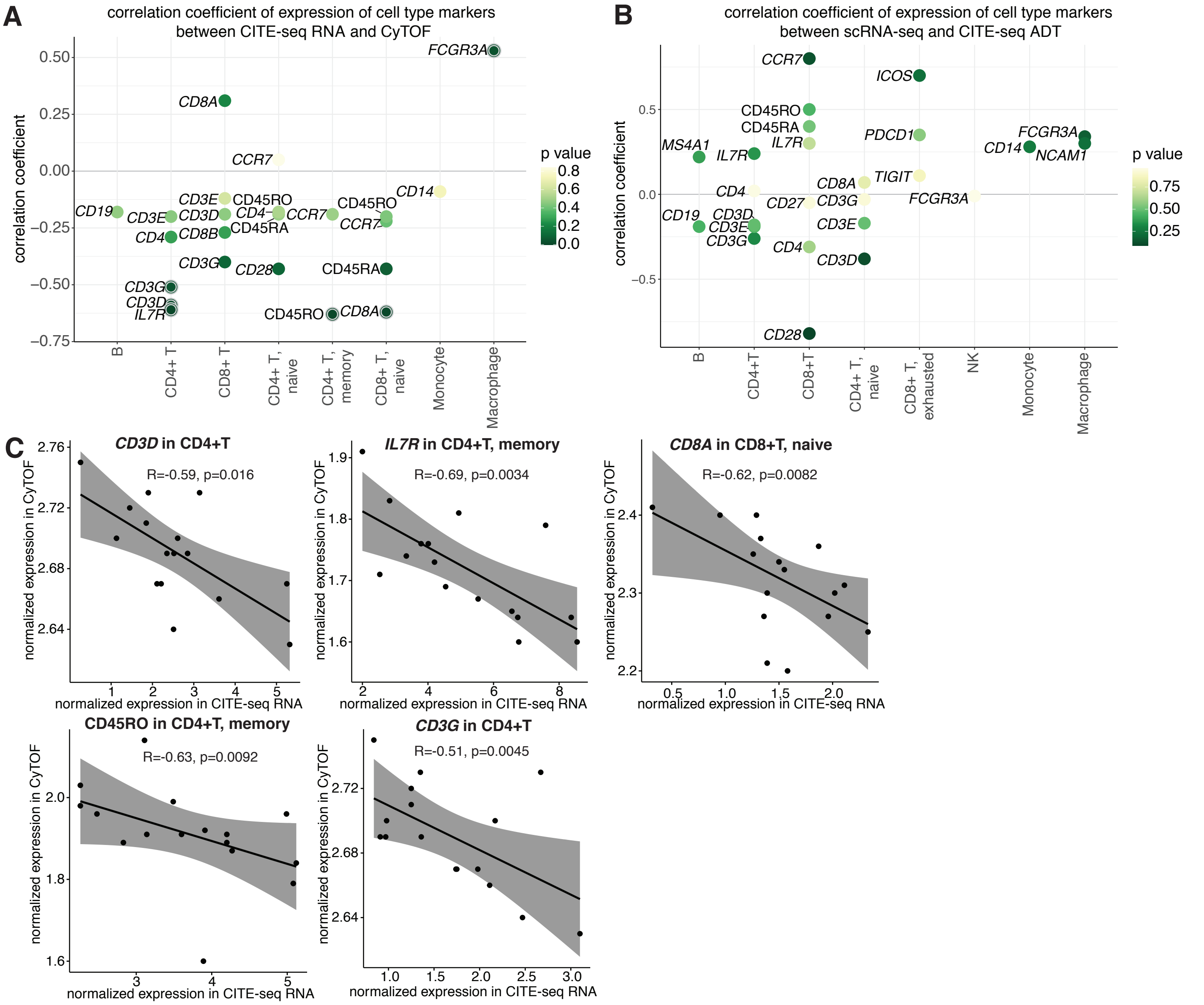
A
correlation coefficient of expression of cell type markers
between CITE-seq RNA and CyTOF
correlation coefficient
p value
FCGR3A
CD8A
CCR7
CD14
CD19
CD3E
CD3D
CD4
CD8B
CD3G
CD45RO
CD45RA
CD28
CD3G
CD3D
IL7R
CD45RO
CD8A
B
CD4+ T
CD8+ T
CD4+ T, naive
CD4+ T, memory
CD8+ T, naive
Monocyte
Macrophage
B
correlation coefficient of expression of cell type markers
between scRNA-seq and CITE-seq ADT
correlation coefficient
p value
CCR7
ICOS
CD45RO
CD45RA
IL7R
PDCD1
FCGR3A
CD14
NCAM1
MS4A1
IL7R
CD8A
TIGIT
CD4
CD27
CD3G
FCGR3A
CD19
CD3D
CD3E
CD3G
CD3E
CD4
CD3D
CD28
B
CD4+T
CD8+T
CD4+ T, naive
CD8+ T, exhausted
NK
Monocyte
Macrophage
C
CD3D in CD4+T
R=-0.59, p=0.016
IL7R in CD4+T, memory
R=-0.69, p=0.0034
CD8A in CD8+T, naive
R=-0.62, p=0.0082
CD45RO in CD4+T, memory
R=-0.63, p=0.0092
CD3G in CD4+T
R=-0.51, p=0.0045
normalized expression in CyTOF
normalized expression in CITE-seq RNA

FIGURE S2

Fig. S2

Correlation of expression of canonical cell type markers across different modalities.

A) Spearman correlation coefficients of cell type markers between transcriptional level expression from CITE-seq and protein level expression from CyTOF. Each dot represents a marker gene and the color of the dot represents the p value of correlation. Markers are highlighted with an outer circle if the p value is less than 0.05.

B) Spearman correlation coefficients of cell type markers between transcriptional level expression from scRNA-seq and protein level expression from CITE-seq. Each dot represents a marker gene and the color of the dot represents the p value of correlation. Markers are highlighted with an outer circle if the p value is less than 0.05.

C) Scatter plots showing examples of negative correlation of expression of cell type marker genes between transcriptional level expression from CITE-seq and protein level expression from CyTOF. MMRF_1505 and MMRF_2251 were excluded due to lack of cell populations of interest based on CITE-seq measurement.
